# Ten simple rules for a mom-friendly Academia

**DOI:** 10.1371/journal.pcbi.1011284

**Published:** 2023-08-10

**Authors:** Esther Sebastián-González, Eva Graciá, Alejandra Morán-Ordóñez, Irene Pérez-Ibarra, Ana Sanz-Aguilar, Mar Sobral

**Affiliations:** 1 Department of Ecology, University of Alicante, Alicante, Spain; 2 Department of Applied Biology, Miguel Hernández University, Elche, Spain; 3 Centro de Investigación e Innovación Agroalimentaria y Agroambiental (CIAGRO-UMH), Miguel Hernández University, Orihuela, Spain; 4 CREAF, Facultad de ciencias y biociencias, Bellaterra, Barcelona, Spain; 5 Department of Agricultural Sciences and the Environment, University of Zaragoza, Zaragoza, Spain; 6 AgriFood Institute of Aragón, Zaragoza, Spain; 7 Animal Demography and Ecology Unit, IMEDEA CSIC-UIB, Esporles, Spain; 8 Department of Biology, University of Balearic Islands, Palma, Spain; 9 Facultade de Bioloxía, Universidade de Santiago de Compostela, Santiago de Compostela, Spain; Carnegie Mellon University, UNITED STATES

## Abstract

Women (and all gender-discriminated people) are underrepresented in science, especially in leadership positions and higher stages of the scientific career. One of the main causes of career abandonment by women is maternity, with many women leaving Academia after having their first child because of the career penalties associated with motherhood. Thus, more actions to help scientific moms to balance family and academic work are urgently needed to increase representation of women and other gender discriminated people in Academia. Besides mothers, these rules may also benefit other groups such as mothers-to-be, fathers, caregivers, and women in general. Increasing women representation in science, including mothers, is critical because equality is a fundamental right, and because more diverse working environments are more productive and get to more optimal solutions. Here, we describe 10 simple rules that can be adopted in Academia to halt the abandonment of scientific careers by women after motherhood. We strongly encourage their implementation to increase gender diversity and equality in science.

## Author summary

Although there is an increasing awareness of the importance of having more diverse scientific communities, the number of women in science is still low. A large proportion of the women who leave their academic career do it because of problems with reconciliation when they become mothers or try to. Therefore, there is a need for more family-friendly academic institutions. Here, a group of moms in science propose 10 simple actions that can be adopted by Academia to directly support mother scientists, (and, indirectly, families in general) and avoid losing them from Academia. We use the term “mothers” for simplicity, but it includes all the groups whose gender identity has an intersectional situation with “maternity,” such as people giving birth (moms and trans dads), trying to give birth, or being primary caregivers of children. Finally, the implementation of the rules may also be beneficial for other researchers (mothers-to-be, fathers, caregivers, and women in general), thus helping to create more diverse Academia.

## Introduction

There is a leaking pipeline in science, technology, engineering, and mathematics (STEM) disciplines that has led to a clear underrepresentation of women in most scientific fields [1–3]. Even for those undergrad and graduate programs where women enroll in similar proportions compared to men, the proportion of women tends to decrease while advancing in the scientific career, and they are especially missing from leadership positions (e.g., [4,5]). Women suffer from systemic and unconscious biases in recommendation, hiring, and tenuring, as well as in recognition and visibility [6–8]. In addition, science led by female scientists is systemically more rejected and less cited than work led by male scientists [9], women are less invited to editorial boards [10,11], receive lower salaries [12], less funding [13], less awards [14], and less lab space [15]. Promoting the presence of women, including mothers, in science is critical not only because equality is a fundamental right for all humans, but also because more diverse working environments are known to be more productive and to get to more optimal solutions [16–18]. For example, a greater female representation in the stock market has been linked to excess returns and higher corporate profitability [18]. Thus, it is imperative to put in place more policies and implement actions to make the academic system more friendly and fair for mothers.

One of the main causes of women dropping a scientific career may be related to family, care-taking, and maternity issues [7,19]. For example, Colwell and McGayne [20] found that about 50% of US female scientists leave science after maternity. Some men leave science too, 23% of those with children versus 16% of those childless, but this number is similar to the number of childless women who abandon science careers (24%, data from around 4,000 scientists in the United States; [21]). This agrees with the differences in daycare duties between male and female scientists: 42.5% of male scientists but only 15.5% of females have a partner taking care of the children during the day [22]. Also, women scientists have a lower average number of children than their male counterparts [23], probably linked to delaying motherhood while pursuing academic careers [24], with potential consequent problems in fertility [25]. In other words, family formation may be one of the main factors that determines why women are systemically underrepresented in Academia.

So, why are moms leaving Academia in particular? Scientists often work long hours in a highly competitive environment (e.g., [26,27]). Therefore, an increase in family duties after motherhood conflicts with the expectations for an academic to be fully devoted to work [28,29]. Taking a career break can have multiple negative effects on women’s career advancement (e.g., [30]); thus, even if available, some women do not use or fully use their maternity leaves for fear that their career will be penalized for it [31,32]. Similarly, only 30% of the eligible faculty in US universities used the tenure probationary period extension policy for parents because of fear of backlash [33].

We use the term “mothers” across the manuscript for simplicity, but it includes all the groups for which their gender identity has an intersectional situation with “maternity” (moms, trans dads, nonbinary parents, etc.). Also, this paper focuses on moms because, as shown above, they are the ones who leave Academia with the highest frequency [21]. More importantly, moms (and trans dads) are the ones who get pregnant, breastfeed, and may have important mental and physical problems because of seeking and carrying out pregnancy and maternity. In heterosexual couples, moms are more likely than dads to take care of sick children or participate in school activities [34,35]. Thus, the effect of parenthood is stronger on mothers.

Here, we describe simple 10 rules (Table 1) that scientists, lab and department heads, scientific institutions, and funding agencies can adopt to curb the abandonment of academic careers by women because of the career penalties associated with motherhood and consequently increase the representation of women in Academia. Importantly, many actions taken to benefit moms will also benefit childless women in science, scientific dads, and caregivers in general, so, in each rule, we identify all potential beneficiaries of its implementation. We also want to underline that we are not asking for a preferential treatment for mothers over other groups with special needs (e.g., scientists with medical issues) and that we encourage that similar actions are developed and implemented for these other groups, considering their specific requirements. Research institutions are making increasing efforts to adopt some of these measures, and several of them have already been implemented but are far from a universal implementation. We strongly encourage their widespread implementation to increase gender diversity and equality in Academia.

**Table 1:** Ten simple rules for a mom-friendly Academia and specific actions that scientists, labs and department heads, scientific institutions, and funding agencies (i.e., stakeholders) can take to achieve them, and potential beneficiaries of such actions.

Rule	Some actions	Stakeholders	Beneficiary
1. Support women during pregnancy	• Technical support to the woman for field and lab work • *Policies to facilitate flexible working hours, remote working, and short-term leave periods if needed • *Clear communication with research institutions and colleagues	• Institutions • Heads • Scientists	• Pregnant people
2. Promote paid parental leaves and respect their use	• Provide paid parental leaves • *Account for leaves in selection processes and eligibility criteria for grants and research positions • *Respect how leaves are used	• Funding agencies • Institutions • Heads • Scientists	• Parents
3. Promote daycare and breastfeeding facilities	• Create affordable daycare and breastfeeding facilities at work and at scientific meetings	• Institutions	• Pregnant people • Parents
4. Organize common lab activities during school times	• *Define the schedule for common lab activities considering the needs of all lab members • *Allow remote working or participation in seminars and meetings	• Institutions • Heads • Scientists	• Parents
5. Give flexibility in working schedules	• *Provide flexibility in working time and/or location • *Allow remote working or participation in seminars and meetings	• Institutions • Heads • Scientists	• All scientists
6. Adapt teaching for parents with little kids	• Provide teaching releases • *Give parents priority to select teaching schedules at school times • *Allow online teaching under some specific circumstances	• Institutions • Heads • Scientists	• Parents
7. Support moms’ academic careers	• *Extend at least 18 months per child the window of eligibility for fellowships and grants for mother scientists • *Waive the geographic mobility requirement in fellowships and grants for mother scientists • Create specific grants after long career breaks	• Funding agencies • Institutions	• Pregnant people
8. Take care of mental health	• Prevent mental health problems through, for example, free counseling available at the workplace • *Set policies to limit work during off-office hours • *Promote family–work balance	• Institutions • Heads • Scientists	• All scientists • Parents
9. Disseminate and enforce protocols against discrimination and harassment	• Create, disseminate, and enforce policies against harassment and discrimination in every research institution	• Institutions • Heads	• All scientists
10. Do not overload women	• *Ensure that the amount of administrative work is equitable	• Funding agencies • Institutions	• Women • Minorities

* Actions that are free of monetary cost.

## Rules

### Rule 1: Support women during pregnancy

Many researchers, especially in the field of experimental sciences, have lab experiments to set or follow up, or their research involves fieldwork close to home or in remote locations that can be physically demanding. In addition, laboratory work may involve the use of chemicals and solvents, radiation, or contact with pathogenic agents, for example, whose handling is incompatible with pregnancy. In all cases, safety for the woman (and trans men) and the baby should come first. Therefore, institutions like the National PostDoc Association of America recommend that women who are trying to become pregnant contact the environmental health and safety office of their institutions as soon as possible and consult their doctor to assess potential risks [36].

It is important to be aware of and handle these risks from the very early stages of pregnancy, when the fetus is more vulnerable [37]. To minimize risk issues, the provision of technical support to the researcher is key. A technician could carry out the experimental/field work, while the pregnant woman can shift toward more desktop/computer-based tasks (e.g., writing, analyzing data, literature review) [38]. We acknowledge that not all labs/groups might be able to support the woman by hiring further personnel, in which case support by research institutions, and clear communication with colleagues will be key to ensure that the research is on track and that teaching expectations are met. For example, by agreement, lab colleagues could take over the lab/field experiments (or part of them) in exchange for desk work or a counter situation in the future.

Besides specific risks related to lab/fieldwork, pregnancy is a physically demanding condition, where tiredness is the norm and health issues might arise. Flexible working hours and the possibility of remote working (see rule #5) might also be favorable during pregnancy. Among other things, flexibility in the hours and place where work is done will facilitate attendance at prenatal checks or the distribution of working hours to periods when productivity is higher. As an example, many women experience physical discomfort at certain times of the day and will work more efficiently when those symptoms subside (see rule #5). In either case, work planning during the months of pregnancy and in preparation for the months of parental leave is key to ensuring that becoming pregnant does not take a toll on women’s professional academic careers or their mental and physical health. This situation urges a fluid communication with colleagues, students, and supervisors [38].

The above recommendations are not limited to pregnancies reaching full term but also apply to other circumstances related to motherhood that are frequently invisible, such as fertility treatments, miscarriages, or adoptions. The delay in maternity in favor of career development [24] and the overoptimistic expectations in fertility [39,40] lead many scientists to face infertility problems when trying to become pregnant [25]. Pursuing fertility treatments often causes psychological and physical discomfort and requires multiple absences from work, adding to the overall distress of the treatment [41]. Workplace policies addressing the needs of scientists undergoing fertility treatments or experiencing a miscarriage could reduce the negative psychological symptoms and prevent possible penalties that these events may have on academic careers (e.g., acknowledging the right to a grief leave after a reproductive loss [42]; see also rule #8).

### Rule 2: Promote paid parental leaves and respect how they are used

Parental leaves correlate with beneficial effects on parents and children regarding their mental and physical health. As an example, maternity leave decreases the risk of postpartum maternal depression [43]. In addition, it can lead to a reduction in mother and infant rehospitalizations while improving breastfeeding success [43]. However, parental leave policies greatly vary among industrialized countries. By 2022, all countries in the Organisation for Economic Cooperation and Development (OECD) had paid maternity leave policies, with the only exception being the US [44]. The length of the covered period for mothers ranges from 12 fully paid weeks in Mexico to more than 80 weeks in Estonia or Romania. Complementing this, long paternity leaves (like in Japan, >60 weeks) allow balanced parenting investments, although this is an unresolved matter for most countries [44]. Academic institutions should recognize the necessity of these leaves in selective processes for jobs or grants and support the application of parents through extended eligibility periods and selection quotas, which may be longer/higher for those scientists experiencing pregnancy, single parents, or scientists experiencing multiple parenthood (see rule #7).

Long leaves are desirable but slow down or sometimes halt the development of academic careers. Until a more inclusive Academia is achieved, many parents, especially mothers, decide to work partially during parental leave. In addition, scientific work has an important vocational component, and some parents are happy maintaining the relationship with their ongoing projects and lab teams. For example, they might like to attend some activities, like seminars, or get involved in important decisions with long-term consequences, like applying for project grants. However, other parents may want to totally disconnect from their work and focus on their babies during this especially intense period of their lives. “Respect” is the word. Family-friendly teams should respect any decision in this regard. Communication before and after birth is essential to know what parents want or can do.

### Rule 3: Promote daycare and breastfeeding facilities at the work place

Modern lifestyles have resulted in higher levels of isolated parenting [45], and scientists often migrate from their familiar communities in pursuit of employment opportunities. Having children close to the office reduces commuting times and facilitates short visits to breastfeed babies if desired. Breastfeeding and daycare facilities in the workplace help to reconcile breastfeeding, childcare, and work schedules and also create social connectivity among employees’ families, which is especially important for mother and child well-being [46]. Daycare facilities need not only to be present, but it is also important to ensure that any worker at the institution is able to use them. This includes having enough vacancies and affordable prices. Also, breastfeeding facilities should be minimally equipped and as many as needed so everybody could reach one in a few minutes’ walk.

These social networks and support to reconcile work and family life can be further maintained through summer schools or camps, especially to cover school holidays. For example, some of the author’s institutions organize summer school camps for the workers’ children during the month of July, when the university is open, but schools are closed. We recommend institutions to enhance familiar work environments that lower stressful daily routines. Finally, we also encourage the inclusion of breastfeeding and daycare services during conferences and scientific meetings to facilitate the attendance of scientists with small children. Many ideas on how to make a conference family-friendly can be found in [47], including on-site daycare facilities and financial support for them.

### Rule 4: Organize all lab common activities during school times

The usually rigid school schedules (from daycare to kindergarten and primary school) critically complicate balancing family and lab work. This complication becomes especially dramatic when programmed lab activities such as meetings, experiments, or fieldwork do not overlap with school times. Our recommendation is to start the academic year by defining with all the lab members the times of the day and days/weeks of the year in which meetings, workshops, and other lab common activities can be scheduled so that these accommodate lab members’ needs to the maximum extent. It can be a challenge to address the schedules of different schools (e.g., public, charter, and private schools) and educational stages and the untimely requirements of international projects. For the later, allowing remote working (details in rule #5) might be a potential solution. Scientific institutions should implement regulations so that these practices are allowed and encouraged.

### Rule 5: Give flexibility in working schedules

Common activities associated with children such as kindergartens’ and schools’ fixed check-in and check-out times, and medical visits, among others, can overlap with parents’ working hours. Moreover, when babies start preschool, they tend to be sick frequently and require at-home care, which also overlaps with working hours. To manage these situations, flexible working systems can be a practical solution. Flexibility in working time and/or location is one of the main measures established by universities, enterprises, and research centers to improve the work–family life balance [48]. In some countries with rich economies and advanced gender policies, such as Iceland, flexible work schedules in Academia are the norm [49]. In others, policies toward flexible work hours and/or online working are increasing, especially after the lessons learned during the COVID-19 pandemic [50]. In this sense, online participation at scientific meetings is currently common, and even online teaching is a possible option at universities with primary face-to-face classes. The pandemic has demonstrated that research and teaching can be done in different and “more flexible” ways. However, flexible work schedules are not implemented everywhere, and there is still a demand for academics to create a family-friendly environment [51].

In general, where established, academics recognize flexible working as a positive and valuable measure to balance family and work life [48,49,52]. However, this flexibility also implies the lack of temporal and spatial limits for working activities and could also increase workload in a highly competitive environment such as Academia [53]. Many of us, during our academic life, have been working (or expected to work) 7 days a week, at nights, and during holidays. By having flexible work hours and working at home, academics can experience “being at work” all the time, which can increase stress and work–family conflicts [54], a fact that affects women more than men [52,55]. Moreover, having a flexible job can also increase the tasks of academics, especially when single or when their partners’ jobs lack such flexibility and all the “family tasks” are concentrated on the academic parent. Although this issue could affect either academic parent similarly, several studies have provided evidence that mothers are more affected (e.g., [49,56]), being the gender that culturally has acquired a higher responsibility for domestic tasks and childcare [57]. Finally, we would like to highlight that a flexible working schedule will also benefit caregivers, individuals with mental or other health issues, and scientists in general [58].

### Rule 6: Adapt teaching for parents with little kids

Teaching can be an important task that takes many work hours for researchers in universities and some research institutions. High teaching loads have been demonstrated to lower research outputs [59], and the latter are generally the most valued when it comes to promoting a scientific career [60,61]. Here, we propose 3 measures to help parents with young children achieve a better balance in both their teaching and research duties.

First, provide teaching releases. The first year after the arrival of a baby is difficult for parents because babies require a lot of attention, especially for moms, who need to recover from pregnancy and delivery. For several years, young children still demand a lot of time from their parents. A very good initiative would be to provide, a total teaching release for the first year after a baby is born for moms, and partial teaching releases for both parents during the following 3 years after childbirth. Partial teaching releases for parents are already implemented in some institutions such as University of Alicante (Spain) where parents get a 30-hour discount in their annual teaching loads for 3 years. It is important to complement this initiative with compensations for the number of teaching hours in the CVs of Academics who have used these teaching releases.

Second, give parents with little kids priority to select teaching schedules during school times. Classes at universities are often taught outside school hours, with many universities having two turns of classes, one in the morning and one in the evening, to adapt to students who work. But daycare for young children outside school times is expensive and often inaccessible.

Last, allow online teaching under some specific circumstances. As remote working can also help reconcile work and family life, we encourage universities to allow online teaching for parents with young children when required by personal situations. As for rule #5, we also encourage to open these rules to other scientists with caregiving duties.

### Rule 7: Implement positive actions to support moms’ academic careers

According to the Gender Equality Commission of the Council of Europe, “positive action” is defined as a temporary strategy that allows to correct those discriminations that are the result of practices or social systems, developing the principle of equal opportunities, to correct inequalities. The structural vulnerability that unfairly affects mother’s scientific trajectories can be alleviated through “positive action” measures that compensate for this structural inequality and are also protected by law (at least at the European Policy level, as far as we know). The implementation of positive measures to encourage mother scientists to continue with their scientific career is imposed as an obligation in the current situation.

Examples of positive actions to help mother scientists include the following: (i) Extend for a few years per child the window of eligibility for fellowships and grants for mother scientists. Although academic women may delay as long as possible the moment of conceiving, the initial part of the scientific career is the hardest and most competitive and this stage coincides, biologically, with the care of young children. Also, women experience stronger physical effects because of pregnancy, postpartum, and breastfeeding. In fact, European Research Council has implemented this since many years ago, and there is an increase of 18 months per child in the potential time to be able to apply for some government support in Europe, specific for women (or people giving birth).

(ii) Waive the geographic mobility requirement to apply to fellowships and grants for mother scientists. Some postdoctoral fellowships require geographic mobility (i.e., moving to a different country from the one where you get your PhD), which is especially hard for women with children, often impossible. For example, to apply for a prestigious European Marie Skłodowska Curie grant, the researcher must not have resided or carried out his/her main activity (work, studies, etc.) in the host organisation’s country for more than 12 months in the 3 years immediately prior to the call deadline.

(iii) Create specific grants or project calls for mothers and other researchers with long career breaks. Long career breaks after maternity or other causes (e.g., caregiving, health issues) often end with women being forced to leave Academia. Implementing specific grants for researchers experiencing these long breaks may give them the chance to come back to the scientific track.

### Rule 8: Take care of mental health

Life and work in Academia is very stressful [62] with deadlines and evaluations happening almost continuously. The levels of anxiety can grow exponentially during parenthood and silent motherhood situations (e.g., fertility treatments, miscarriages) that can lead to distress, depression, and anxiety among other mental health problems and affect motivation, productivity, and overall career progression [42]. Moreover, factors such as the impostor syndrome (more prevalent in women [63]) or the absence of female role models in Academia [64] do not contribute to improving the mental health of women in science. Taking care of mental health is urgently needed and will benefit the entire scientific community.

Knowing the high risk of suffering from anxiety, depression, and other mental problems is the first step to take care and improve mental health in Academia. Actions such as the implementation of specific policies to minimize solutions based on a case-by-case basis (e.g., grief policies [42]; see rule #1), the promotion of training in mental health issues for supervisors, and of measures to foster a good family–work balance, including the application of the rules proposed here, are key to reduce burnouts and mental health problems and to increase diversity in science.

### Rule 9: Disseminate and enforce the protocols against discrimination and harassment

Despite significant improvements toward gender equity in Academia in the last few years, academic work models remain unsupportive of parenthood [65] since the latter clashes with the expectations for an academic to be fully devoted to work and have no family commitments or career interruptions [28]. Therefore, parenthood, especially motherhood, is a common basis of discrimination in the academic environment. Mothers in the early stages of their careers are more susceptible to harassment because of their lower position on the academic ladder, with work power being a significant predictor of discrimination and harassment for women [66]. To protect mothers and other groups commonly discriminated against in Academia because of race, ethnicity, or class, policies against harassment and discrimination should be in place in every research institution. Not only should such policies exist, but it is also important to ensure that they are enforced (e.g., through auditing). These policies should develop clear protocols for dealing with situations of discrimination and harassment and provide training to employees, so they are aware of, able to identify, and know how to report common forms of discrimination that affect them or their colleagues.

### Rule 10: Do not overload women

Even if it is not an issue specific to mothers, we feel the need to call for research institutions to avoid overloading women with administrative work. Many universities and research centers are adopting policies to increase women representation (e.g., [67]). However, some of those policies penalize women instead of helping them. For example, it is a common demand that women represent at least 50% of the members of hiring, dissertation, and thesis committees. There is also an increasing demand for women as manuscript and grant reviewers. In fields where women are underrepresented, as happens in many STEM areas, this practice increases the hours of unpaid unrecognized work that women must contribute. For example, if a department has 3 women and 7 men, and all the committees need to be egalitarian, the women will need to be present in many more of those unrecognized administrative activities. Positive actions to include more women are needed, but extra care is needed to avoid penalizing more than helping them. Thus, institutions should put in place regulations to ensure that the amount of administrative work is equitable, irrespectively of gender. This rule can also be applied to other underrepresented minorities facing a similar situation.

## Concluding remarks

To sum up, all stakeholders in Academia can do their bit for a more friendly environment for families. While some of our rules require financial support, others are free (see Table 1) and should be the norm. Implementing these rules will have many benefits, as it will increase the compatibility of working in Academia with raising a family, ultimately reducing the proportion of parents leaving the academic career, especially mothers.

We strongly encourage that our rules be considered to increase women’s permanence in Academia. However, it is important to note that most of these recommendations are not specific for universities and research centers, and some of them, such as offering flexible working schedules or avoiding meetings outside school times, can be easily adopted in other ambits, like enterprises or public administrative positions. Thus, we also submit a request for more mom and family-friendly working environments in general. In addition, children with disabilities or health problems have caring demands that extend for longer time periods than those considered here, and these measures should be extended for parents under such circumstances.

Although we tried to be as general as possible, each culture and country has adopted different family-friendly actions, from countries where maternity leave is unpaid to others with paid leaves for both parents for more than a year [38]. Also, the scientific career varies (e.g., [68,69]), some countries use a tenure-track system, while in others, professors are selected after an exam; pre- and postdocs are workers with full rights (i.e., paid holidays, insurance…) in some countries, while in others, they receive scholarships without any benefit. Thus, some of these rules may be broadened and adapted to the different policies and rules in each country. Finally, these rules could also be adapted to caregivers and scientists with specific situations, such as long-term health issues, and promote a more friendly and fair work environment in Academia.
